# Metabolic syndrome in women with previous gestational diabetes

**DOI:** 10.1038/s41598-021-90832-0

**Published:** 2021-06-02

**Authors:** Karsten Kaiser, Michael Festersen Nielsen, Ervin Kallfa, Greta Dubietyte, Finn Friis Lauszus

**Affiliations:** 1 Sygehus Sønderjylland, Aabenraa, Denmark; 2 Herning Hospital, Herning, Denmark; 3 Department of Gynecology and Obstetrics, Aabenraa Hospital, Kresten Phillipsensvej 15, 6200 Aabenraa, Denmark

**Keywords:** Cardiology, Diseases, Endocrinology

## Abstract

To evaluate the incidence and timing of the diagnosis of metabolic syndrome in a cohort of Danish women after a pregnancy with gestational diabetes (GDM) to estimate the optimum time for preventative actions in relation to metabolic syndrome (MetS). In this follow-up study, 435 women were included from a consecutive cohort with prior history of GDM. Data on dyslipidemia, hypertension and other cardiovascular disorders (CVD) were extracted from the electronic patient journal. Any antidiabetic, cardiovascular and cholesterol-lowering medicine was ascertained in the national prescription database. Similarly, any blood test taken was evaluated. We defined a patient having MetS if the criteria of the WHO based definition of diabetes or impaired glucose regulation were met. Further, we added as alternative for glucose intolerance, a glycosylated hemoglobin (HbA1c) > 44 mmol/mol or the former level ≥ 6.5%. Further, dyslipidemia, lipid lowering medications, BMI > 30 kg/m^2^ or antihypertensive treatment were used. For MetS outcome, diagnosis or medication for CVD was registered. All women were followed for median 5.7 years (range 0; 9). The incidence of MetS was 28%. Thirteen percent of these qualified already within one year after pregnancy for the diagnosis of MetS. Postpartum MetS was detected after a median of 3 years (range 0; 7 years); further, 36 (8%) had been diagnosed with manifest diabetes after pregnancy. The diagnosis of postpartum MetS was strongly associated with the prevalence of manifest diabetes. Six years after pregnancy the rate of metabolic syndrome was more than tripled (25 vs. 89%, no DM vs manifest DM, RR: 6.7; 95% CI 2.7–17, p < 0.001). At 40 years the MetS rate nearly tripled if manifest DM was diagnosed (26 vs. 78%, no DM vs. manifest DM, RR: 3.3, 95% CI 1.8–6, p < 0.001). We found that GDM and later on manifest DM in women increase the risk of metabolic syndrome. There seems to be a window of opportunity before the early thirties where it would be especially beneficial to begin preventive efforts in women with GDM.

## Introduction

Gestational diabetes mellitus (GDM) occurs in 3–4% of pregnancies in Denmark and the rate apparently increases during the last 10 years. This is attributed to the obesity pandemic and escalating overweight in pregnant women^1,2^. Currently GDM is diagnosed by an oral glucose tolerance test (OGTT) using the criterion of ≥ 9 mmol/l two hours after intake of 75 g of glucose^2^. An increased risk was reported in these women for development type 2 diabetes mellitus (T2DM) and cardiovascular disorders (CVD) years after delivery, which is suggestive for subsequent metabolic syndrome (MetS), too^3–7^. This syndrome represents a cluster of risk factors including abdominal obesity, raised triglycerides, reduced HDL cholesterol, hypertension, and raised fasting plasma glucose^8^. WHO concluded in 2010 that MetS is not just a diagnosis, but a pre-morbid condition needing intervention through lifestyle changes and possibly medication to reduce CVD^9^.

Further, MetS is part of what is called non-communicable diseases, including heart disease, stroke, cancer, diabetes and chronic lung disease, which are collectively responsible for almost 70% of all deaths worldwide. According to the WHO, almost three quarters of all deaths of non-communicable disease and 82% of the 16 million people who died prematurely occur in low- and middle-income countries^10^.

In this report, we hypothesized that GDM is one step on the way to MetS but little is known about the magnitude of the association and timing of its development to the full syndrome in order to identify a window of opportunity for prophylaxis. Thus, detecting GDM in pregnancy could be a way of early identification of the risk of developing MetS, maintaining good health and avoiding T2DM and CVD later in life. Several studies investigate this association between GDM and the individual components of MetS, but only few of these studies involve a Scandinavian population and a complete follow-up on a pre-existing cohort^11–13^.

The aim of this study is to evaluate the incidence of MetS and subsequent cardiovascular disorders in a cohort of women with prior GDM in order to register morbidity and estimate the optimum time for preventative actions in relation to MetS. We have previously reported on the subsequent development of manifest diabetes in the same cohort^14^.

## Material and methods

We performed a follow-up on a cohort study including all women diagnosed with GDM between 2011 and 2016. The Regional Ethics committee and Data Protection Agency gave approval for the study with permission to access data anonymized without the written consent of patients (Nos. 1-16-02-824-17, 1-16-05-825-17, 1-16-02-180-17, 1-16-02-378-19), which was conducted in accordance with the Helsinki Declaration and the guidelines for Good Clinical Practice. The name of the ethics committee is “De videnskabsetiske Komitéer for Region Midtjylland”, Skottenborg 26, DK-8800 Viborg, Denmark, Phone: + 45 7841 0183, E-mail: komite@rm.dk.

GDM was diagnosed in 3% of the women delivering at the hospital, based on threshold glucose values at 120 min of venous plasma or capillary whole blood ≥ 9 mmol/l or ≥ 10 mmol/l in capillary plasma. The screening indications were maternal pre-pregnancy body mass index ≥ 27 kg/m^2^, family disposition of diabetes, previous GDM, multiple pregnancy, previous macrosomia (birth weight ≥ 4500 g), stillbirths, polycystic ovary syndrome or glycosuria. Beside medical nutrition therapy, all women with GDM had ambulatory visits where a medical endocrinologist and a diabetes nurse assessed the glycemic regulation every second week from diagnosis. The current WHO based definition of diabetes or impaired glucose regulation is at OGTT including fasting plasma blood glucose levels of ≥ 6.1 mmol/l, 2-h glucose values ≥ 7.9 mmol/l^15^. Further, we added as alternative for glucose intolerance a glycosylated hemoglobin (HbA1c) ≥ 44 mmol/mol or the former level ≥ 6.5%.

Hospital charts data were collected from the electronic journals together with paraclinical data and medications and prescriptions in current or previous use. We registered age, height, pre- and post-pregnancy weight, non-Danish origin, smoking, comorbidity, and parity. Further, we collected data on screening indication, date of diagnosis of GDM, gestational age and neonatal outcomes including length, birth weight, and head circumference. Blood samples were available on-line from 2006 and onwards from hospitals, general practitioners and private specialists. These were registered: Blood lipids (HDL, LDL, and total cholesterol, and triglycerides (TG)) before and after pregnancy, HbA1c, mean glucose, 2-h glucose at 75 g OGTT and fasting blood glucose at the time of diagnosis, during pregnancy (last sample before giving birth), postpartum (≤ six months after delivery) and the most recent glucose evaluation. Further, hospital registers were used to systematically gather the following data from each woman: (1) Previous or current diagnosis related to metabolic syndrome such as obesity (for MetS diagnosis), CVD like hypertension for outcome, and DM for MetS diagnosis. (2) Previous or current prescribed medication on following indications: Hypertension and angina pectoris for outcome; dyslipidemia or DM for MetS diagnosis. (3) Blood samples since 2006 of total-, LDL-, and, HDL cholesterol and triglycerides. (4) Height and most recent weight registered.

This study is based on the WHO criteria and accordingly the diagnosis of MetS was: Glucose intolerance or diabetes together with two of the other components: (1) Raised arterial pressure ≥ 160/90 mmHg4. (2) Raised plasma triglycerides (≥ 1.7 mmol l⫺1; 150 mg/dl) and/or low HDL-cholesterol (< 1 mmol/ l; 39 mg/dl in women) (3) Central obesity (females: waist to hip ratio > 0.85) and/or BMI > 30 kg/m^2^ (4) Microalbuminuria (urinary albumin excretion rate ≥ 20 μg /minor albumin:creatinine ratio ≥ 20 mg/g^15^. The women treated with cholesterol lowering medication were categorized as fulfilling the WHO criteria regardless of the newest blood sample results when the former was abnormal. As for CVD outcome: one woman with angina pectoris and no relevant blood samples available was not classified as having metabolic syndrome; women, with a history of antihypertensive medication or a previous hypertension diagnosis, were regarded as fulfilling the hypertension criterion. We excluded any duplicate, second pregnancies of women delivering more than once during 2011–16 (n = 23). The follow-up therefore contains 435 GDM women.

### Statistical analysis

To test for significant difference between two variable means, the Student’s t-test was applied if data followed a Gaussian distribution. Otherwise, Mann–Whitney’s U-test was used. Proportions were tested with χ2 test and 95% confidence intervals (95% CI) were calculated using http://vassarstats.net/. The continuous variables age, glucose at OGTT (fasting and 2-h glucose), follow-up time were subjected to Kaplan–Meier analysis with MetS diagnosis after pregnancy as group variable. Log- rank test was applied for significance testing. Cox regression analysis was performed on the outcome of MetS with age, BMI, 2-h glucose at OGTT in pregnancy, and parity as continuous covariates and the categorical variables of screening indication of DM in the family history, insulin treatment during pregnancy, and subsequent DM diagnosis. Follow-up times are given as median (range). Statistical analyses were conducted using the statistical software program IBM SPSS Statistics 24. Data are given as mean ± SD if they followed a Gaussian distribution; if not, median and range are indicated. As the level of significance, a two-sided p value of < 0.05 was chosen. Those who emigrated or died since delivery were entered with the date of last contact with the public registry and with known diagnosis at that time. All other women were followed-up by entries in registries on diagnosis, laboratory measurements and prescription used.

## Results

The cohort of 435 women with prior GDM was followed up median 5.7 years (range 0.2;9). Accordingly, 120 (28%) were diagnosed with MetS with a median age of 35.2 (range 21–49), hereof were 16 (13%) normal weight women. Fifteen of the women (3% of all women and 13% of all diagnosed with MetS) qualified already within one year after pregnancy for the diagnosis of MetS. Of the 120 women, 72 had dyslipidemia (17% of all and 60% of all diagnosed with MetS) and 69 (58%) had had a pre-gravid weight ≥ 30 kg/m^2^. Postpartum MetS was detected after a median of 3 years (range 0.2;6.8); further, 36 (8%) had been diagnosed with manifest diabetes after pregnancy.

The women’s pre-pregnancy age, height and parity were similar with respect to MetS (Table 1). However, weight, BMI, and fasting glucose and 2-h glucose at OGTT were higher in women diagnosed with MetS and they were more often treated with insulin during pregnancy for their GDM. Despite insulin treatment the average glucose and HbA1c levels were higher in the women who later developed MetS, We found similar neonatal anthropometrics irrespective of MetS.

**Table 1:** Basal characteristics and pregnancy data of 435 women with GDM by subsequent metabolic syndrome.

	Women with MetS (n = 120)	Women without MetS (n = 315)	All women with GDM (n = 435)	p-value^a^
Age at delivery (years)	32 ± 5.3	31.7 ± 5.1	31.8 ± 5.1	0.42
Pre-pregnancy weight (kg)	88 ± 21	79 ± 18	82 ± 19	0.001
Height (cm)	166 ± 7	166 ± 6.7	166 ± 6.8	0.24
BMI (kg/m^2^)	31.8 ± 6.5	28.9 ± 6	29.7 ± 6.3	0.001
Parity no.	1 (0–4)	1 (0–6)	1 (0–6)	0.26
Family history of diabetes no. (%)	34 (28)	53 (17)	87 (20)	0.01
Etnicity-non-Danish no. (%)	26 (22)	65 (21)	91 (21)	0.9
Fasting BG at diagnosis (mmol/l) ^b^	5.9 (4.6–8.6)	5.4 (4–8.2)	5.6 (4–8.6)	0.001
2-h-value at OGTT (mmol/l)^c^	10.2 (8.8–18.8)	9.7 (7.9–20.8)	9.7 (7.9–20.8)	0.001
Insulin treatment during pregnancy no. (%)	37 (31)	25 (8)	62 (14)	0.001
Mean weekly blood glucoe (mmol/l)	6.7 ± 0.7	6 ± 0.7	6.3 ± 0.8	0.001
HbA1c last measurement (mmol/mol)	41 ± 5	35 ± 5	37 ± 5	0.001
Birth weight (g)^d^	3614 ± 543	3512 ± 495	3541 ± 507	0.065
Gestational age (days)	273 ± 10	274 ± 11	274 ± 11	0.59
Birth length (cm)	52 (42–57)	51 (44–61)	52 (42–61)	0.39
Ponderal index (kg/dm^3^)	26 ± 2.9	26 ± 2.6	26 ± 2.7	0.29
Head circumference (cm)	35 (28–38)	35 (29–38)	35 (28–38)	0.96
Manifest diabetes at follow-up no. (%)	30 (25)	6 (2)	36 (8)	0.001
Cholesterol (mmol/l)^e^	5.2 (2.7–7.6)	5.1 (2.9–8.1)	5.1 (2.7–8.1)	0.48
HDL- cholesterol (mmol/l)	1.15 (0.7–2.2)	1.26 (0.5–3.1)	1.22 (0.5–3.1	0.011
LDL cholesterol (mmol/l)	3.1 (1–5.9)	3.1 (1.2–5.9)	3.1 (1–5.9)	0.74
Triglycerid (mmol/l)	2.24 (0.5–7.2)	1.61 (0.2–5.7)	1.83 (0.2–7.2)	0.001

Data are given as mean ± SD / median (range) or no. (% of column).

*BG* blood glucose.

^a^Women with vs. without metabolic syndrome.

^b^Data on fasting-BG in 172 women.

^c^Data on 2-h-BG in 427 women.

^d^Data on neonatal anthropometrics in 428 women.

^e^Data on lipidemia in 250 women.

At our follow-up we could confirm that in total 404 women (92%) had their glucose measured at various times after delivery: 399 by fasting glucose, 394 by 2-h glucose after OGTT, and HbA1c in 109 women. Only 30% had an OGTT within six months post partum. Of the 36 who were diagnosed with manifest diabetes 14 were treated with metformin and two with insulin and metformin. In 20 women no anti-diabetic medication and prescription could be found in the national registry that covers all doctors and pharmacies.

Similarly, we found that in total 250 women had measurements of lipids. Dyslipidemia was found in 172 women (40% of all) at a median age of 33; 64 women had high TG, 28 low HDL, and 80 both; 20 (12%) women were treated with lipid-lowering medication (simvastatin, n = 9; atorvastatin, n = 10, rosuvastatin n = 1). In seventeen women, dyslipidemia was diagnosed before pregnancy. If manifest diabetes was diagnosed, dyslipidemia was more prevalent and women were more often obese and had had higher fasting glucose during pregnancy (Table 2). More than 95% women who were found with impaired glucose regulation, like those manifest DM, had dyslipidemia in combination with BMI > 30 kg/m^2^; the remaining women had either hypertension/medication for hypertension or microalbuminuria in combination with dyslipidemia.

**Table 2:** Components of the diagnosis metabolic syndrome in 120 women with previous gestational diabetes by subsequent manifest diabetes diagnosis.

	Women with manifest DM N = 30	Women without manifest DM N = 90	p-value ^a^
Age at delivery (years)	31 ± 5.5	32.4 ± 5.1	0.34
BMI > 30 (kg/m^2 ^)	15 (50)	54 (60)	0.39
Dyslipidemia total no. (%)	24 (80)	48 (53)	0.005
Low HDL cholesterol no. (%)	22 (73)	41 (46)	0.007
High TG no. (%)	18 (60)	33 (37)	0.022
BMI (kg/m^2^)	32.3 ± 7.5	31.7 ± 6.2	0.44
Fasting BG at GDM diagnosis (mmol/l)	6.4 (5–8.6)	5.8 (4.6–8.5)	< 0.05
2-h-value at OGTT during pregnnacy (mmol/l)	11.1 (9–15)	10 (8.8–18)	0.16
Insulin treatment during pregnancy no. (%)	19 (63)	18 (20)	0.001
Birth weight (g)	3628 ± 535	3609 ± 548	0.87
Gestational age (days)	271 ± 10	274 ± 11	0.19
Birth length (cm)	52 (48–56)	52 (42–57)	0.66
Ponderal index (kg/dm^3^)	26 (21–30)	26 (21–37)	0.76
Head circumference (cm)	35 (31–38)	35 (28–38)	0.85
Cholesterol (mmol/l)	5.2 (3–7.5)	5.1 (2.7–7.6)	0.89
HDL-cholesterol (mmol/l)	1.1 (0.8–1.8)	1.17 (0.69–2.2)	0.73
LDL cholesterol (mmol/l)	3.1 (1.4–5.9)	3.1 (1–5.7)	0.3
Triglycerid (mmol/l)	2.28 (0.7–7)	2.22 (0.5–7.2)	0.83

Data are given as mean ± SD/median (range) or no. (% of column).

*DM* Diabetes mellitus, *BG* Blood glucose.

^a^Women with vs. without diabetes.

As for outcome, of 20 women (4.6%) with CVD, 17 had hypertension treated with ACE inhibitors (n = 10), beta-blockers (n = 10), and diuretics (n = 3, in combination with the others). Two women suffered a stroke and were diagnosed with valvular fibroelastoma and persistent foramen, respectively, and one woman had transitory cerebral ischemia. The former three and further one women with hypertension were treated with acetyl salicylate or clopidogrel. CVD was suspected in further eight women in whom neither a definitive diagnosis was made after Holter monitoring nor was any treatment instituted. Five women with CVD (25%) had no apparent dyslipidemia and did not qualify for MetS. The incidence CVD was similar in women with DM diagnosis or impaired glucose regulation (data not shown).

Body weight proved to be inadequately registered as most follow-ups were at the general practitioner (data not shown). Pre-gravid 104 (24%) women had a BMI < 25 kg/m^2^, 45% were obese with ≥ 30 kg/m^2^; five women had a gastric bypass surgery after pregnancy; three of them were categorized as having MetS. The rate of metabolic syndrome was 15, 26 and 35% in women with pre-gravid normal weight, overweight and obesity, respectively (p < 0.001).

**Figure 1:**
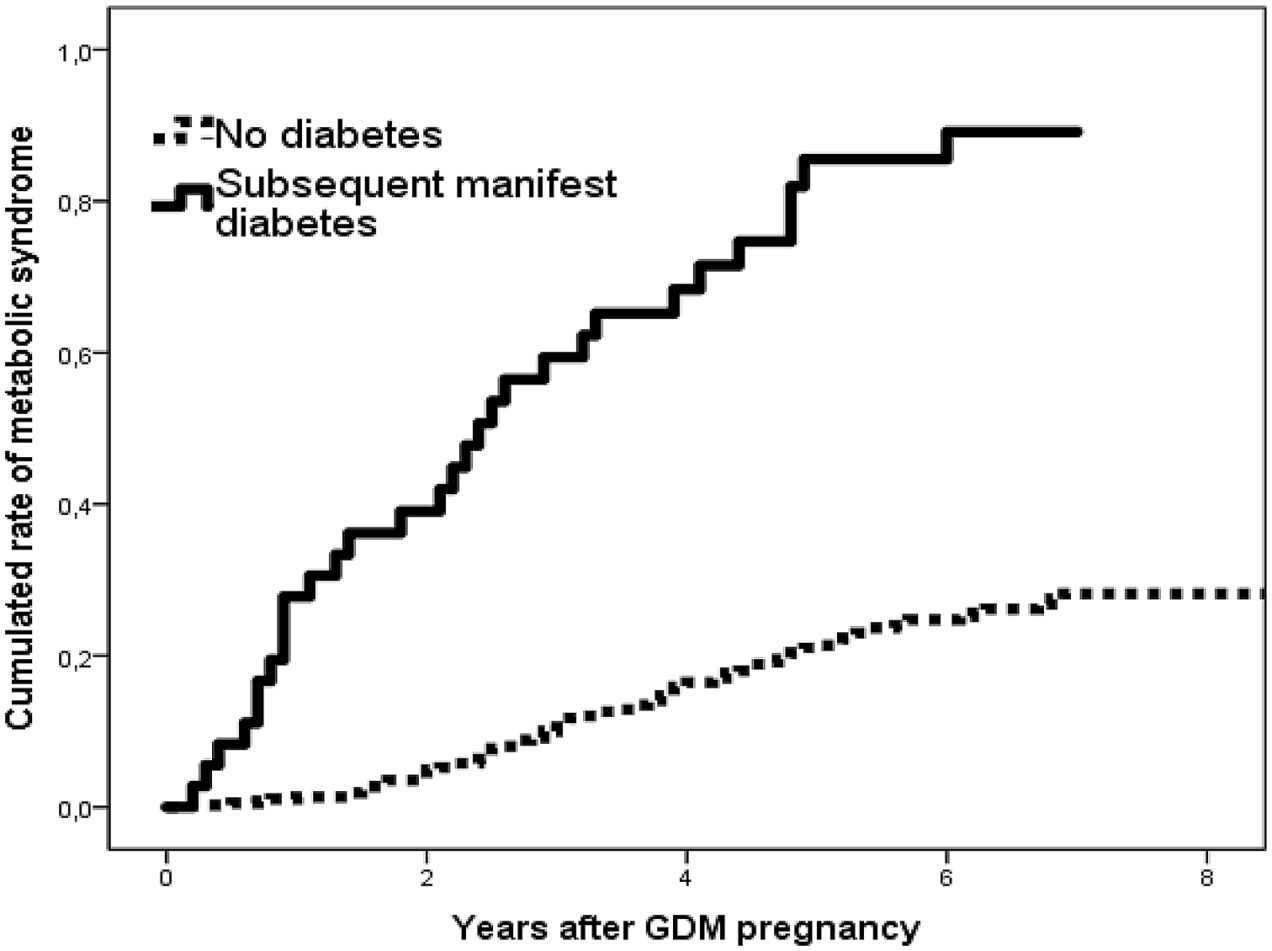
Diagnosis of metabolic syndrome after gestational diabetes in pregnancy by subsequent manifest diabetes. Full line: Women with subsequent diabetes; interrupted line: Women without manifest diabetes. No diabetes vs. manifest diabetes: p > 0.001.

**Figure 2:**
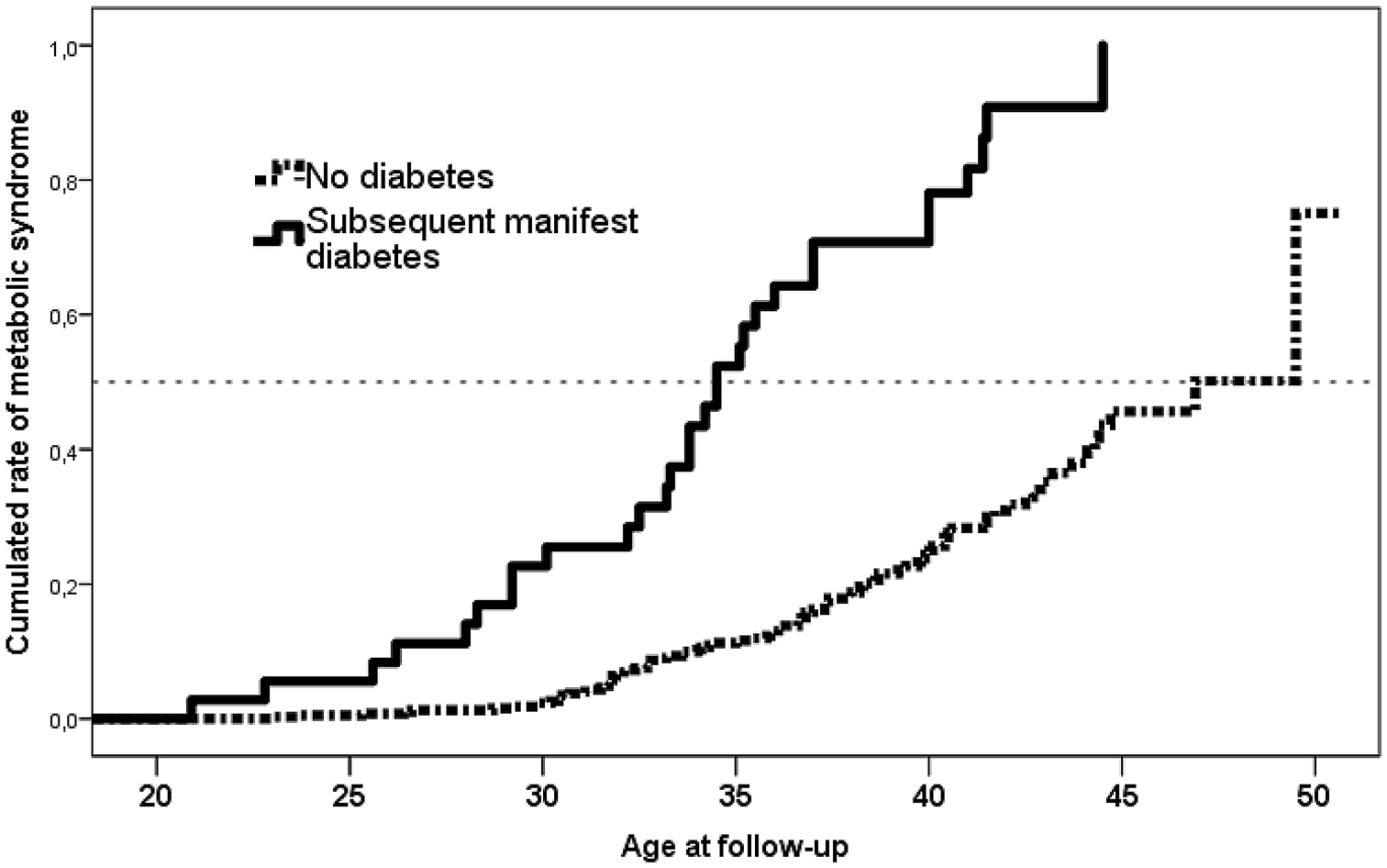
Age at diagnosis of metabolic syndrome by subsequent manifest diabetes. Horizontal line indicating when 50% of women were diagnosed with MetS. Full line: Women with subsequent diabetes; interrupted line: Women without manifest diabetes. No diabetes vs. manifest diabetes: p > 0.001.

The diagnosis of postpartum MetS was strongly associated with the prevalence of manifest diabetes (Fig. 1). Six of 36 women with manifest DM (17%) had no further morbidity indicating MetS. Six years after pregnancy the rate of metabolic syndrome was more than tripled (25 vs. 89%, no DM vs manifest DM, RR: 6.7; 95% CI 2.7–17, p < 0.001). When looking at which age MetS was diagnosed we found at 40 years the rate nearly tripled if manifest DM was diagnosed (26 vs. 78%, no DM vs. manifest DM, RR: 3.3, 95% CI 1.8–6, p < 0.001, Fig. 2). Half of the women with manifest diabetes were diagnosed with MetS at age 34 and those without manifest diabetes at age 46. At regression analysis BMI, insulin treatment during pregnancy and subsequent DM diagnosis was associated with MetS while age, family history of DM and 2-h glucose at OGTT in pregnancy was not.

## Discussion

We found that the timing of when MetS is diagnosed should allow for sufficient time for preventive interventions before significant cardiovascular morbidity takes place. However, we found no difference in CVD with respect to glucose intolerance and manifest diabetes, but still nearly 5% had CVD in the total cohort. The prevention of further risk depends on identification and awareness of the different aspects of MetS: For one, these women had had GDM, secondly, one has to think in weight control and lipidemia at check-up. The outcome of CVD may not come into the general practitioners mind unless non-communicable disease are considered. Still, more than 1/4 of women with previous GDM had MetS at 35 year of age or within nine years after pregnancy. This incidence of metabolic syndrome is somewhat less than reported in previous Scandinavian studies on GDM women: Lauenborg et al. found 40% at median 43 years of age in the Danish capital, 17% 1 year after delivery was found in Western Finland, and 60% before the age of 37 years in the Eastern Finland province^8,11,12^. Again, the maternal weight seemed to be a major determinant as the highest incidences of MetS are found in populations with a high BMI. All the studies confirm that GDM women have a relative risk of 3 for developing metabolic syndrome^8,11,12^. Even when leaving out the maternal weight and just adding dyslipidemia we observed more MetS at a younger age than any of the other studies. Thus, we assume the incidence of metabolic syndrome in our GDM population at least at a similar level. These findings in GDM women are in accordance with several other European studies showing an up to 6 times higher incidence of metabolic syndrome in GDM populations compared to normoglycemic women during pregnancy, using different criteria for metabolic syndrome^12,16–18^. Only one study concluded women have similar metabolic syndrome after GDM as women without GDM^8^. A possible explanation to this finding, contrary to that of other studies, might be that the participation rate of the GDM women was merely 27%.

Definitions of the metabolic syndrome are not unanimous and were suggested by WHO^15^, the European Group for the study on Insulin Resistance (EGIR)^19^, and the National Cholesterol Education Program Expert Panel (NCEP-ATP-III)^20^. In our study, we decided to define metabolic syndrome according to the WHO criteria for comparison with similar Scandinavian studies^11^. In a Spanish study of women with prior GDM, metabolic syndrome was diagnosed in 14% of the women according to the WHO criteria and in 22% of the women when using the NCEP-ATP-III criteria, indicating that the WHO criteria gives a lower incidence than the former^18^. Our study constitutes, therefore, an even conservative estimate of the incidence of metabolic syndrome than studies using the NCEP-ATP-III criteria: This underestimation would apply too, when considering that we miss some data in our cohort and that diagnostic criterion on GDM is ongoing discussed and not uniform.

Our study had some limitations as it can be assumed that the GDM women already fulfilled the requirement of some degree of glucose intolerance in contrast to most ‘normal’ women, who can adequately respond to the increased demand for insulin during pregnancy. The association between prior GDM and development of DM later in life^6^, though, reinforces this assumption. However, it would be more accurate to verify the glucose intolerance post-partum, which on the other hand can be difficult to achieve due to difficulties with compliance and adherence to guidelines. Thus, the follow-up with an OGTT was performed in less than 1/3 of the women within six months after delivery despite national guidelines. Bearing in mind that we find manifest diabetes strongly associated with MetS, looking for the first thing would help find the other. For this, knowing not only the magnitude of the disorder but also the timing this study point at optimum timing when to introduce prevention, screening and medication before onset of manifest symptoms. Due to our access to national registries on medications and laboratory data, we were able to follow-up on the whole cohort unlike the other Scandinavian studies who invited their participants in for assessments losing 40–50% of the original cohorts. This could explain the difference in age in the debut of MetS, nevertheless, it may be a more accurate estimation of the timing of the interval for interventions.

We obviously lack data on men at similar age and some baseline information such as smoking status, dietary and exercise habits, and socioeconomic status, which could be important as a co-variate. Furthermore, it would be beneficial to include a control group without GDM, so we more accurately could estimate the increased incidence of metabolic syndrome in women with GDM. On the other hand, we offer a comprehensive follow-up due to the completeness of the prescription, laboratory and diagnosis databases and a relatively simple approach of using already available data. The catch is whether any follow-up was performed and for most of the women, we did have to rely on the general practitioner’s follow-up or pre-gravid health check-ups on evaluating dyslipidemia. Not all the available blood samples were fasting blood samples but in most cases aberrant blood samples were re-taken at proper circumstances. Body weights proved absent in most cases except from those who later admitted to hospital so the pre-pregnant measurements had to suffice; we assume that most people tend to gain weight with age rather than the opposite. As weight is part of the criterion of MetS it is no surprise that it is associated with MetS and cannot be evaluated as an independent factor.

Only relative few of the women were diagnosed with CVD and we may suspect that if all the women had a proper check-up even more would have been found, adding to the assumption that our findings are even a conservative estimate on the consequences of metabolic syndrome in our population. Furthermore, among the women who developed metabolic syndrome, three percent had documented dyslipidemia short time prior to their GDM condition and, therefore, confirming the biological plausible link of diabetes and lipid metabolism; what remains dubious is which causes the morbidity leading to CVD.

In concordance, the antenatal glucose at OGTT and insulin requirement during pregnancy, too, were predictors of metabolic syndrome, but most likely in first line are determinants for subsequent manifest diabetes pointing at beta cell dysfunction^13,21,22^. Some studies found pre-pregnancy overweight and obesity to be even more important predictors of metabolic syndrome^12,22^, especially when combined with a fasting glycemia^22^. Anyway, if the ultimate prevention goal is CVD then women with GDM appear to be at high risk of any of the diagnosis of dyslipidemia, DM, overweight, and hypertension as part of the non-communicable diseases. The rise of these are driven by primarily four major risk factors according to other studies: Tobacco use, physical inactivity, the harmful use of alcohol and unhealthy diets, all of which our study was unable to address.

The time line analysis showed that 26–78% will develop metabolic syndrome at the age of 40 years, depending of their diabetes diagnosis (Fig. 2). We believe it to indicate an interchangeable correlation between glucose and lipid metabolism. In line with this is our observation that ¾ of the women with MetS were diagnosed with overweight or had dyslipidemia; however, the neonates did not weigh more or grow disproportionately in the MetS group rather than in those women with manifest diabetes later on.

Whether prevention of one metabolic disorder will prevent the other, too, remains debatable. A hint that it may be worthwhile is the observation that the rate of MetS was postponed more than 10 years in women with glucose intolerance compared to those with manifest diabetes (Fig. 2). In any case, one must be concerned about the high percentage of women with metabolic syndrome in a rather young age; our finding suggests a potential health benefit in preventive efforts among prior GDM before their early thirties or at any point when diagnosed after this age. In one study on preventive efforts, 35% of women did not attend the postpartum revision visit and GDM women with important predictors of metabolic syndrome were more frequently lost to follow-up^22^. This is important to consider when prevention strategies are devised and implemented.

## Conclusion

We found GDM associated with high risk of metabolic syndrome and later on manifest DM in women increases the risk of metabolic syndrome. There seems to be a window of opportunity before and at the age of 30 where it would be especially beneficial to begin preventive efforts in women with GDM. Antenatal glucose measurements and BMI should be used to select women with a higher risk of developing metabolic syndrome, so the condition with its subsequent cardiovascular outcomes can be sought out more effectively and prevented through lifestyle modifications, weight loss and medical intervention when needed. Larger studies are needed to evaluate the age where the incidence of metabolic syndrome rises substantially. Furthermore, evaluation of prevention strategies needs to be performed.

## Abbreviations

CVD: Cardiovascular disease
DM: Diabetes mellitus
GDM: Gestational diabetes
MetS: Metabolic syndrome
OGTT: Oral glucose tolerance test
RR: Relative risk
T2DM: Type 2 diabetes mellitus
